# Blood Exosomes Have Neuroprotective Effects in a Mouse Model of Parkinson's Disease

**DOI:** 10.1155/2020/3807476

**Published:** 2020-11-26

**Authors:** Ting Sun, Zhe-Xu Ding, Xin Luo, Qing-Shan Liu, Yong Cheng

**Affiliations:** Key Laboratory for Ethnomedicine for Ministry of Education, Center on Translational Neuroscience, College of Life and Environmental Sciences, School of Pharmacy, Minzu University of China, Beijing, China

## Abstract

Parkinson's disease (PD) is a common and complex neurodegenerative disease; the pathogenesis of which is still uncertain. Exosomes, nanosized extracellular vesicles, have been suggested to participate in the pathogenesis of PD, but their role is unknown. Here, a metabolomic analysis of serum and brain exosomes showed differentially expressed metabolites between 1-Methyl-4-phenyl-1, 2, 3, 6-tetrahydropyridine hydrochloride- (MPTP-) induced PD mice and control mice, such as oxidized lipids, vitamins, and cholesterol. These metabolites were enriched in coenzyme, nicotinamide, and amino acid pathways related to PD, and they could be served as preclinical biomarkers. We further found that blood-derived exosomes from healthy volunteers alleviated impaired motor coordination in MPTP-treated mice. Results from immunohistochemistry and western blotting indicated that the loss of dopaminergic neurons in substantia nigra and striatum of PD model mice was rescued by the exosome treatment. The exosome treatment also restored the homeostasis of oxidative stress, neuroinflammation, and cell apoptosis in the model mice. These results suggest that exosomes are important mediators for PD pathogenesis, and exosomes are promising targets for the diagnosis and treatment of PD.

## 1. Introduction

Parkinson's disease (PD) is the second most common neurodegenerative disease, affecting approximately 1% of the population aged > 60 years [1, 2]. A meta-analysis of the global data indicated the rising prevalence of PD with age along with some differences in prevalence by geographic location and sex [3, 4]. The first detailed description of PD was made approximately two centuries ago, but the pathogenesis of the disease remains unclear [5]. Its cardinal motor symptoms are bradykinesia, tremor, rigidity, flexed posture, postural instability, and freezing of gait [6, 7]. The crucial pathological feature of PD is the degeneration of dopaminergic neurons in the substantia nigra pars compacta (SNpc), a reduction in dopamine content in the striatum (Cpu), and the appearance of Lewy bodies [5, 6], which are aggregated by abnormally folded *α*-synuclein. Misfolded *α*-synuclein is the most abundant protein inclusion that can categorize PD with other neurodegenerative diseases. PD results from a complicated interplay of genetic and environmental factors, such as cigarette smoking and caffeine [8–10]. However, we cannot treat PD by modifying genetic or environmental risk factors. Neuroinflammation also contributes to PD pathology. In the early stages of diseases, astrocytes and microglia are both involved in the clearance of extracellular debris or release of nutrients and anti-inflammatory factors, which might aid the survival of neurons [11, 12]. However, activated microglia can release harmful reactive oxygen species, nitrogen species, and proinflammatory cytokines [13, 14]. Prolonged overactivation of microglia will exacerbate the death of dopaminergic neurons in the nigra [15, 16]. Accumulating evidence indicates that oxidative stress plays a key role in all forms of PD [17]. Studies of young PD patients have revealed that elevated oxidative stress is an important trait of the early disease stages and occurs before severe neuronal loss [18]. Exosomes, the smallest extracellular vesicles (diameter range 50-150 nm), are released by the fusion of multivesicular endosomal bodies with the plasma membrane [19, 20]. Exosomes can be released both *in vitro* and *in vivo* from a variety of nerve cells, such as neurons [21], microglia [22], and astrocytes [23]. Exosomes carry noncoding RNA such as miRNA, lncRNA, and circRNA, along with proteins and lipids [24, 25], and mediate intercellular and interorgan communication. In PD, exosomes secreted by neurons have been found to carry pathogenic proteins such as *α*-synuclein and are transmitted to other neurons or glial cells, leading to the further spread of misfolded *α*-synuclein and the occurrence of neuroinflammation [26–28]. There is reason to believe that the release and transmission of exosomes can dynamically reflect the pathological changes in cells in inaccessible areas such as the brain [29]. Some studies have demonstrated that the expression of proteins and RNAs in exosomes derived from the sera and cerebrospinal fluid (CSF) of PD patients significantly differ from those of healthy people [30]. The studies by Shi et al. [31] and Stuendl et al. [32] found significant differences in the exosomal *α*-synuclein in the plasma and CSF exosomes in PD patients and controls. The study also found a large increase in the number of exosomes in the CSF of PD patients [32]. Therefore, exosome has been suggested as a promising target for the diagnosis of PD [33]. Given the close relationship between dopamine loss and the metabolism of amino acids such as tyrosine, exosomal metabolomics analysis could receive more attention for predicting disease or discovering new potential biomarkers. However, there are no detailed studies on exosomal metabolites in relation to PD. In addition, signal molecules carried by exosomes may also affect gene expression in target cells. Many studies have indicated that exosomes derived from multiple cells may have therapeutic effects in neurological diseases such as stroke [34, 35], ischemia-reperfusion injury [36], traumatic brain injury [37, 38], and Alzheimer's disease [39]. Our latest study found that serum exosomes from major depressive disorder patients caused depressive-like behavior in healthy mice, and serum exosomes from healthy volunteers alleviated the depressive-like behaviors in unpredictable mild stress-treated mice [40]. We hypothesize that exosomes may also have potential predictive and therapeutic effects in PD. In this study, we are aimed at utilizing widely-targeted metabolomics technology to analyze the serum and brain exosomal metabolite differences between healthy and 1-Methyl-4-phenyl-1, 2, 3, 6-tetrahydropyridine hydrochloride- (MPTP-) induced PD mice, with the aim of identifying potential biomarkers of PD. In addition, we are aimed at exploring whether “normal” exosomes protect against PD.

## 2. Materials and Methods

### 2.1. Preparation of Exosomes

All volunteers were recruited from Minzu University of China (Supplementary Table 1). The blood exosome isolation and validation methods were performed as previously reported [41]. Briefly, exosomes were isolated from the diluted blood using a qEV column and concentrated with a 30 K MWCO PES protein concentrator-Vivaspin® (Sartorius, Gottingen, Germany). The concentrated exosomes were then resuspended in 200 *μ*l phosphate-buffered saline (PBS) for further analysis.

### 2.2. Widely-Targeted Metabolomics

Widely-targeted metabolomics detection and quantification were conducted by the metabolomics provider METWARE on serum and brain samples from healthy control and MPTP-induced PD mice. Serum and brain exosome isolation was performed as described previously [41, 42]. Detailed methods are provided in Supplementary file 1.

### 2.3. Animals and Treatment

Ten-week-old male C57BL/6 mice (25 ± 2 g) were obtained from Vital River Laboratory (Beijing, China) and were housed at 24 ± 1°C and 50 ± 1% humidity under a 12 h light/dark cycle and provided with *ad libitum* access to a standard diet and drinking water. Animal procedures (including the euthanization method) were approved by the Animal Care and Use Committee of the Minzu University of China (ECMUC2019001AO). MPTP (Macklin, Shanghai, China) was used to generate the PD model. The mice were randomly divided into three groups: the control, MPTP, and exosomes+MPTP groups. The mice in the control group were intraperitoneally injected with saline. The MPTP and exosomes+MPTP groups were injected with MPTP (20 mg/kg, dissolved in saline) every 2 h for a total of four doses over an 8 h period in 1 day. The mice in the exosomes+MPTP group were caudal vein injected with exosomes (0.2 ml per mouse). The first injection was delivered 1 h before the first injection of MPTP, and thereafter once every 3 days for a total of four injections.

### 2.4. Behavior Test

The rotarod test was used to assess neurological impairment such as motor coordination and balance and was performed on days 1 and 11 after MPTP injection. The protocol followed was as previously reported [43]. Before MPTP injection, mice were pretrained for 3 days at 10 rpm until they were able to remain on the rod for at least 90 s. For the formal test, a rotating drum was accelerated from 4 to 40 rpm over 5 min. Each trial continued until the mice were unable to remain on the rod without falling off. The latency for the mice to fall from the rotarod was recorded (*n* = 3).

### 2.5. Brain Immunohistochemistry

After the rotarod test, mice were euthanized with pentobarbitone and perfused with saline. The brains were removed, postfixed in 4% paraformaldehyde overnight at 4°C, and then soaked in 20% and 30% sucrose and processed for cryoprotection. The frozen brains were cut into 40 *μ*m coronal sections using a freezing microtome and stored in cryoprotectant (30% ethylene glycol, 30% sucrose, and 0.02 M PB) at -20°C until use. Brain sections were washed in PBS and treated with 3% H_2_O_2_, then blocked and perforated with 10% goat serum and 0.3% Triton X-100. The sections were then incubated overnight with primary antibodies TH (CST, Boston, MA, USA) at 4°C, followed by incubation with secondary antibodies which were goat anti-rabbit (1 : 400) for 1 h at 25°C. Sections were mounted on gelatin-coated slides, then dried, dehydrated, and coverslipped. The histological images were examined using a bright-field microscope.

### 2.6. Western Blot Analysis

The substantia nigra and striatum were isolated from the brain and homogenized with RIPA buffer. The protein concentration of the lysates was determined by a BCA Protein Assay Kit and mixed with 4× loading buffer and boiled at 95°C for 5 min. Proteins were separated by sodium dodecyl sulfate–polyacrylamide gel electrophoresis and then transferred to a nitrocellulose membrane. The percentage of the SDS-PAGE gel was 10% for tyrosine hydroxylase (TH) and actin and 12% for Bax and Bcl-2. The blotted membrane was blocked with 5% skim milk for 1 h and then incubated overnight with corresponding primary antibodies at 4°C, followed by incubation with corresponding secondary antibodies for 1 h at 25°C, and subsequently visualized with an enhanced chemiluminescence reagent. The secondary antibodies were goat anti-rabbit (1 : 10,000) and goat anti-mouse (1 : 10,000) from CST.

### 2.7. Quantitative Real-Time Polymerase Chain Reaction

Total RNA from substantia nigra and striatum was, respectively, extracted with Trizol reagent. A PrimeScript RT reagent kit was used to synthesize first-strand cDNA. Then cDNAs were quantified by real-time polymerase chain reaction on LightCycler® 96 (Roche). GAPDH was used as a reference gene for analysis. Primers were synthesized by Sangon Biotech (Shanghai) Co., Ltd.

### 2.8. Oxidative Stress Marker Level/Activity Measurement

The MDA level and total SOD activity in the serum were measured by a colorimetric assay kit (Jiancheng Bioengineering Institute, China) according to the manufacturer's instructions.

### 2.9. Statistical Analysis

Data are presented as means ± standard deviation. Statistical significance (*p* < 0.05) was analyzed by one-way ANOVA or t-test with GraphPad Prism 7.

## 3. Results

### 3.1. Differentially Expressed Metabolites between Control and MPTP Mice

**Figure 1:**
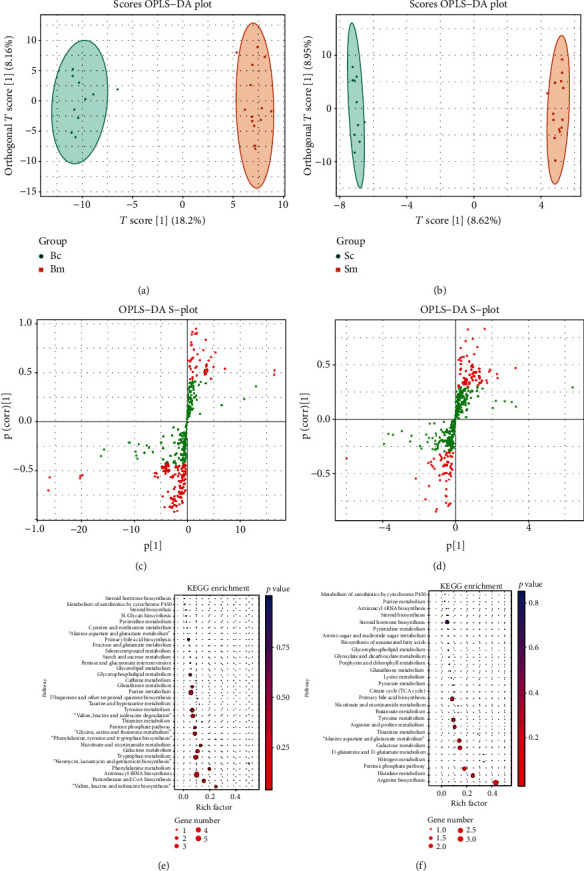
Widely-targeted metabolomics analysis was used to evaluate the differences of metabolites in the brain between control and PD model mice. Analysis of 14 Parkinson's disease mice and 10 control mice. A Score OPLS-DA Plot of metabolites in the brain (a) and serum (b). An OPLS-DA S-Plot of metabolites in the brain (c) and serum (d). Statistics of KEGG Enrichment in the brain (e) and serum (f).

A widely-targeted metabolomics analysis was used to evaluate the differences in exosomal metabolites in the serum and the brain in healthy control and MPTP-induced PD mice. In this study, a total of 433 metabolites were detected in four groups: serum control (Sc), serum MPTP (Sm), brain control (Bc), and brain MPTP (Bm). A Score OPLS−DA Plot and an OPLS-DA S-Plot of Bc vs. Bm and Sc vs. Sm are presented in Figure 1. The metabolic data were analyzed according to the OPLS−DA model, and the Scores OPLS−DA Plot was drawn to illustrate the previous differences in each component.

The Variable Importance in Projection (VIP) of the OPLS-DA model and *p* value as determined by a Mann–Whitney *U*-test were used to screen the differential expression of metabolites. A VIP of ≥1 and *p* value of < 0.05 were considered to indicate differentially expressed metabolites. The results of the differential metabolism screening are provided in Supplementary Table 1. In the serum samples, 69 metabolites were found to be differentially expressed between the control group and MPTP group. In the brain tissue, 148 metabolites were found to be differentially expressed (Supplementary Tables 2 and 3). Additionally, 25 differently expressed metabolites were found in both serum and brain tissue (Table 1).

We next used the KEGG database to annotate the differentially expressed metabolites for potential biological functions. According to the KEGG pathway enrichment diagram, pathways such as the tyrosine metabolic pathway, purine metabolism, and glutamate metabolism pathways were found to be significantly enriched for differentially expressed metabolites in the serum and brain tissue (Figure 1).

### 3.2. Serum Exosomal Metabolites as Potential Biomarkers for PD

**Figure 2:**
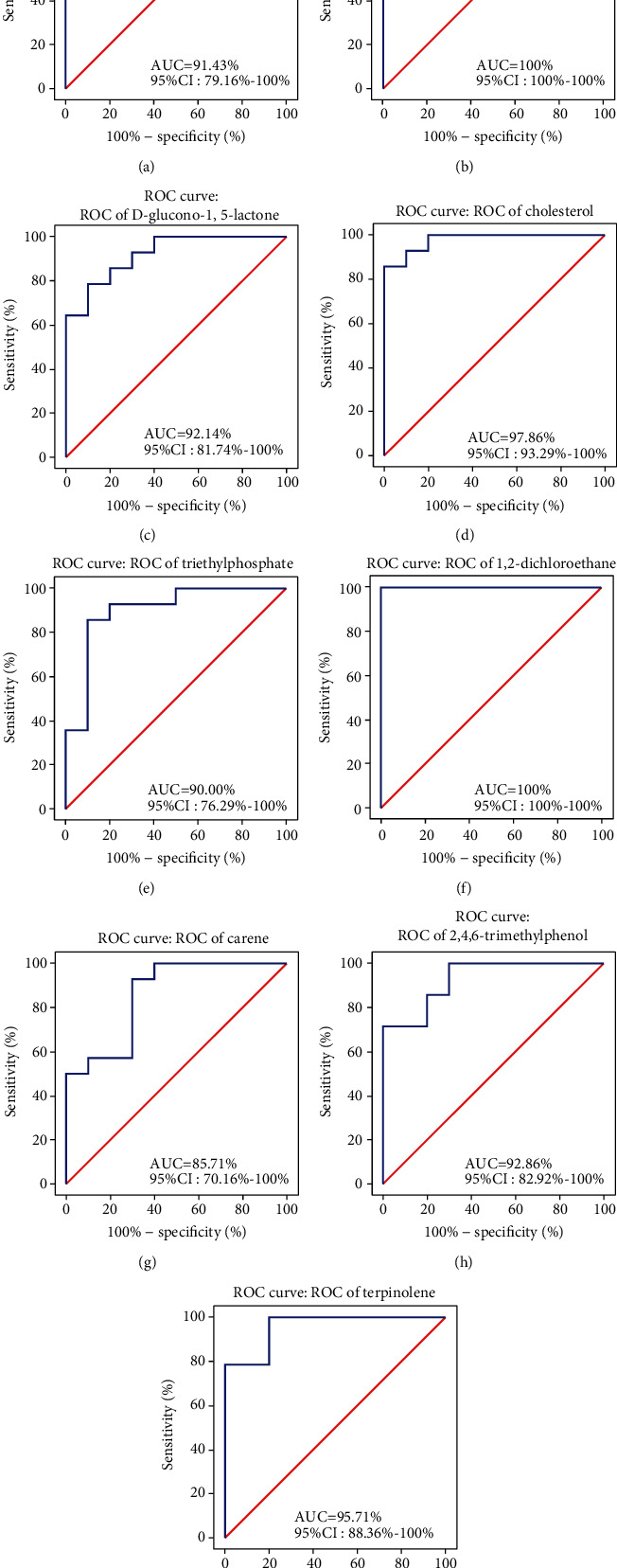
Serum exosomal metabolites as biomarkers for PD. ROC curves of Phenyllactate (a), (S)-(-)-2-hydroxyisocaproic acid (b), D-dlucono-1,5-lactone (c), cholesterol (d), triethyl phosphate (e), 1,2-dichloroethane (f), carene (g), 2,4,6-trimethylphenol (h), and Terpinolene (i) in serum exosomes of MPTP mice and controls. Results were analyzed with 10 controls and 14 MPTP mice.

Receiver operating characteristic (ROC) curves were utilized to evaluate the accuracy of the metabolites in the serum for potentially differentiating MPTP mice from controls. We performed ROC curve analysis using the nine metabolites that contributed most to the differentiation of MPTP mice and controls. As indicated in Figure 2, serum exosomal phenylacetate (Figure 2(a), area under the curve [AUC] = 91.43%, 95% confidence interval [CI]: 79.16-100%), (S)-(-)-2-hydroxyisocaproic acid (Figure 2(b), AUC = 100%, 95% CI:100-100%), D-glucono-1,5-lactone (Figure 2(c), AUC = 92.14%, 95% CI: 81.74-100%), cholesterol (Figure 2(d), AUC = 97.86%, 95% CI: 93.29-100%), triethyl phosphate (Figure 2(e), AUC = 90%, 95% CI: 76.29-100%), 1,2-dichloroethane (Figure 2(f), AUC = 100%, 95% CI: 100-100%), carene (Figure 2(g), AUC = 85.71%, 95% CI: 70.16-100%), 2,4,6-trimethylphenol (Figure 2(h), AUC = 92.86%, 95% CI: 82.92-100%), and terpinolene (Figure 2(i), AUC = 95.71%, 95% CI: 88.36-100%) were found to have good to excellent performance to discriminate between MPTP mice and controls. This suggests that the above nine metabolites are likely to be potential biomarkers for PD.

### 3.3. Effects of Blood-Derived Exosomes from Healthy Volunteers on Motor Ability in PD Model Mice

**Figure 3:**
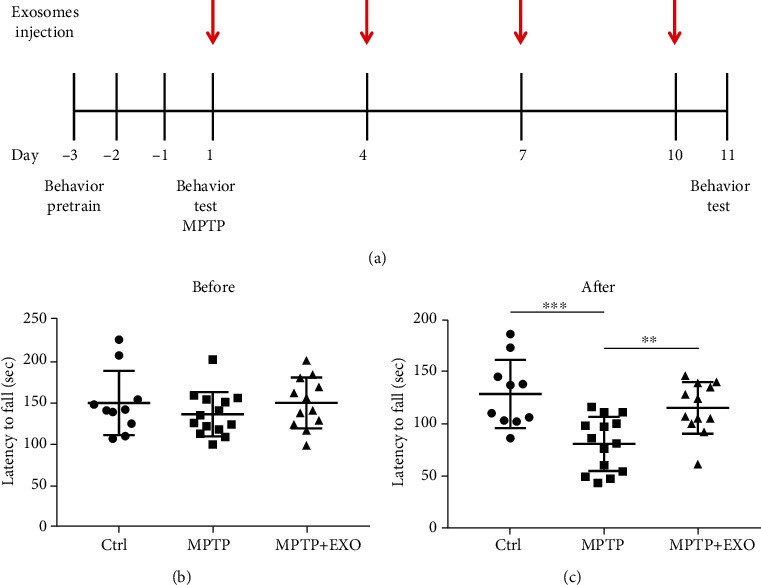
Exosomes improve behavioral performances in MPTP-induced mice. Protocol for MPTP administration, exosome administration, and behavioral test in Parkinson's disease model mice (a). Rotarod test for mice before MPTP injection (b), and after MPTP injection (c). All values are means ± SD, *n* = 10 − 14, ^∗∗^*p* < 0.01, and ^∗∗∗^*p* < 0.001.

The rotarod test was used to evaluate the motor coordination and balance of mice. The experimental design is illustrated in Figure 3(a). After pretraining and before MPTP injection, there was no significant difference in the latency of the mice in each group, indicating that the motor capacity of the mice in each group was similar (Figure 3(b)). On day 11, the rotarod test results indicated that MPTP treatment significantly decreased the latency and exosome treatment significantly improved the latency (Figure 3(c)) (*p* < 0.001).

### 3.4. Effects of Blood-Derived Exosomes from Healthy Volunteers on Dopamine Neurons in MPTP Mice

The improvement in motor dysfunction in exosome-treated MPTP-treated mice led us to hypothesize that exosomes may protect dopaminergic neurons from MPTP-induced injury. To test this hypothesis, we assessed whether exosomes prevent MPTP-induced neuronal damage. TH is a rate-limiting enzyme in dopamine biosynthesis and a well-known marker of dopaminergic neurons. The level of TH-positive neurons in the SNpc and CPu is recognized as an indicator of the severity of dopaminergic neuronal damage in MPTP-injured animals.

**Figure 4:**
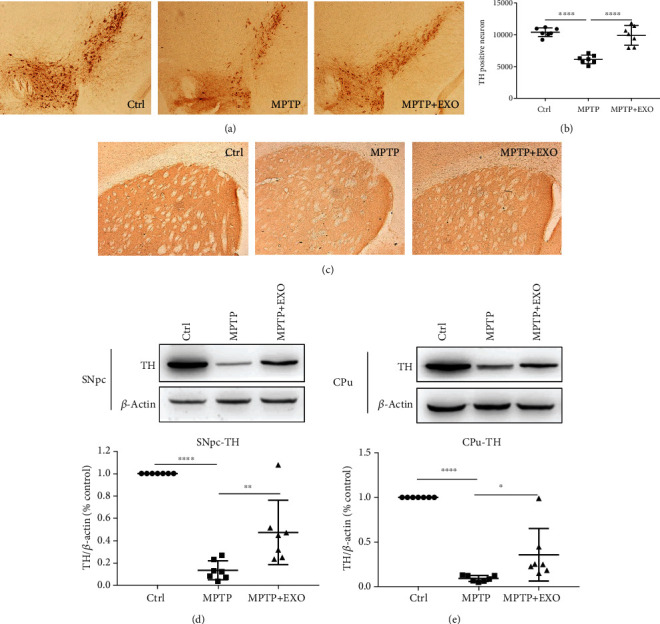
Exosomes prevent tyrosine hydroxylase (TH) loss in SNpc and CPu. Representative photomicrographs of TH-positive neurons in the SNpc region (a), quantitation of TH-positive neurons in SNpc (b), and representative photomicrographs of TH-positive neurons in the CPu region (c). Representative immunoblots and quantification of TH in the SNpc (d) and in the CPu (e). Error bars represent the means ± SD, *n* = 7, ^∗^*p* < 0.05, ^∗∗^*p* < 0.01, ^∗∗∗^*p* < 0.001, and ^∗∗∗∗^*p* < 0.0001.

The immunohistochemical results in the SNpc indicated that the number of TH-positive neurons was significantly reduced in the MPTP group compared to that in the control group (*p* < 0.0001). Exosome treatment almost completely reversed this reduction (*p* < 0.0001) (Figures 4(a) and 4(b)). Similar results were also reflected in the immunohistochemical staining of the CPu (Figure 4(c)). To further confirm these findings, we measured TH protein levels in the SNpc and CPu by western blot. Compared to the control group, TH protein expression was significantly reduced in both the SNpc and the CPu in the MPTP group (*p* < 0.0001), whereas exosome treatment significantly alleviated this reduction (*p* < 0.05) (Figures 4(d) and 4(e)).

### 3.5. mRNA Levels of Inflammatory and Anti-Inflammatory Factors in the SNpc and CPu

**Figure 5:**
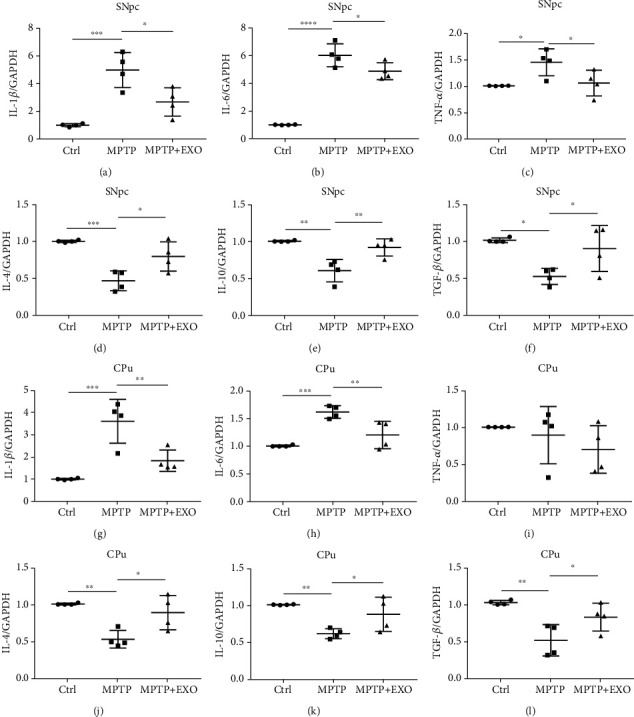
Exosomes attenuate neuroinflammation in SNpc and CPu. Data from qRT-PCR analyses of L-1*β* (a), IL-6 (b), TNF-*α* (c), IL-4 (d), IL-10 (e), TGF-*β* (f) in SNpc, and L-1*β* (g), IL-6 (h), TNF-*α* (i), IL-4 (j), IL-10 (k), TGF-*β* (l) in CPu. Error bars represent the means ± SD, *n* = 4, ^∗^*p* < 0.05, ^∗∗^*p* < 0.01, ^∗∗∗^*p* < 0.001, and ^∗∗∗∗^*p* < 0.0001.

Neuroinflammation is one of the key pathogenic features of PD. In this study, we examined inflammatory and anti-inflammatory cytokine mRNA expression in the SNpc and CPu. The results revealed that the mRNA levels of interleukin (IL)-1*β*, IL-6, and tumor necrosis factor- (TNF-) *α* were significantly increased and those of IL-4, IL-10, and transforming growth factor- (TGF-) *β* were significantly decreased by MPTP treatment in the SNpc (*p* < 0.05). Exosome treatment significantly reduced the mRNA levels of IL1*β*, IL-6, and TNF-*α* and improved the mRNA levels of IL-4, IL-10, and TGF-*β* (*p* < 0.05) (Figures 5(a)–5(f)). Similar changes in IL-1*β*, IL-6, IL-4, IL-10, and TGF-*β* were observed in the CPu (*p* < 0.05) (Figures 5(g)–5(l)). These results indicate that neuroinflammation caused by MPTP could be relieved by exosome treatment.

### 3.6. Effects of Blood-Derived Exosomes from Healthy Volunteers on Oxidative Stress and Apoptosis

**Figure 6:**
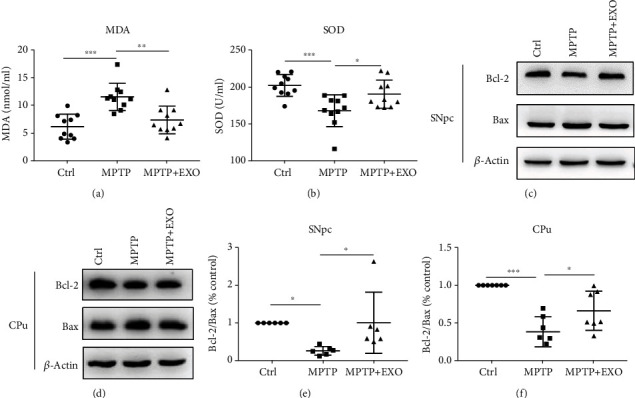
Exosomes alleviate oxidative stress injury in serum and the apoptosis in both SNpc and CPu. Effects of exosome treatment on the changes of MDA (a) and total SOD (b), *n* = 10. Representative bands of Bcl-2, Bax, and quantitative analysis results of Bcl-2/Bax in SNpc (c, e) and Cpu (d, f), *n* = 7. All values are means ± SD, ^∗^*p* < 0.05, ^∗∗^*p* < 0.01, and ^∗∗∗^*p* < 0.001.

Oxidative stress has been implicated in the etiology of PD. The serum malondialdehyde (MDA) and superoxide dismutase (SOD) levels were measured to assess the degree of oxidative stress injury and antioxidative ability. The results indicated that, compared to the control condition, MPTP treatment significantly increased the MDA level, and exosomes markedly alleviated this change (*p* < 0.05) (Figure 6(a)). Similarly, the SOD level was significantly reduced by MPTP but increased by exosomes (*p* < 0.05) (Figure 6(b)). In addition, Bcl-2 and Bax were detected, which reflected antiapoptosis and apoptosis in the brain, respectively. The results indicated a significant reduction in Bcl-2 protein expression but an increase in Bax expression with MPTP treatment (*p* < 0.05). Exosomes also upregulated Bcl-2 and downregulated Bax expression (*p* < 0.05). The above results were consistent in the SNpc (Figures 6(c) and 6(e)) and CPu (Figures 6(d) and 6(f)).

## 4. Discussion

The etiology of PD is complex and diverse and involves the interaction of genetic factors and the external environment. The occurrence of the disease is often associated with imbalances in neurotransmitters, lipid and energy metabolism disorders, and mitochondrial dysfunction. Neurotransmitters play a role in signal transduction via metabolic pathways such as release and reuptake. Changes in any of these metabolic pathways may affect central nervous system function. Small changes in endogenous and exogenous factors can be reflected in metabolite levels. Based on metabolomics approaches, metabolites associated with dopamine, purines, amino acids, fatty acids, and polyamines were found to be highly correlated with the progression of PD [44, 45]. Most of such studies are based on CSF and blood analysis, although some have examined other biological samples such as urine, feces, or brain tissue [46]. However, blood (serum or plasma) metabolites do not fully reflect the metabolism in the brain, and CSF samples are difficult to acquire. Therefore, exosomes, which can cross the blood-brain barrier, were used in this study to synchronously assess the metabolic changes in both the brain and the serum. Our results revealed a close relationship between exosome metabolites and PD. In this study, we found significant differences in 13 amino acids and metabolites in brain exosomes. Tyrosine and phenylalanine, which are closely involved in dopamine metabolism, were significantly downregulated in the PD group compared to the control group. Tyrosine is taken up by dopaminergic neurons and catalyzed by tyrosine hydroxylase in the cytoplasm to L-dopa, which is subsequently decarboxylated to dopamine. Phenylalanine also forms tyrosine via phenylalanine hydroxylase. The reduction of tyrosine and phenylalanine reflects the reduction of dopamine, which is one of the most important mechanisms in PD. The decrease in dopamine results in relative hyperfunction of the acetylcholine system. This transmitter disorder is the key to dyskinesia and other abnormal neurological activities. Our findings indicated that in PD mice, glutamic acid, glutamine, and glutamate were significantly reduced in serum exosomes; branched-chain amino acids (leucine, isoleucine, and valine) were reduced in brain exosomes; and alanine was increased in both serum and brain exosomes. Notably, there were significant differences in many lipids and fatty acids in both serum and brain exosomes between PD and control mice. The findings are similar to those of previous studies reporting that these metabolism pathway alterations are all indicative of mitochondrial dysfunction [44, 47]. In addition, decreased adenine in both serum and brain exosomes may be related to their participation in biological oxidation reactions. Uric acid, the final product of purine metabolism, can remove reactive oxygen and reactive nitrogen and reduce oxidative/nitrative stress. These results confirmed that the metabolism of alanine, purine, and branched amino acids and lipids, which are closely involved in mitochondrial function and biological redox homeostasis, play an important role in the progression of PD. Moreover, the metabolites of exosomes were also indicative of alterations to carbohydrate metabolomics pathways. The serum and brain exosomes of PD mice accordantly exhibited a significant decrease in dulcitol and an increase in D-glucono-1, 5-lactone. In brain exosomes, phosphate and glucose were significantly reduced in PD mice. In serum exosomes, galactose and galactopyranoside were significantly increased in PD mice. It is not yet clear why these trends differ, but it may reflect the differential release of exosomes from different parts of the body. These results were consistent with previous results in body fluids [44]. The KEGG database revealed the differential metabolic pathways of phenylalanine and tyrosine metabolism, tryptophan metabolism, purine metabolism, branched-chain amino acid metabolism (leucine, isoleucine, and valine), etc. Other previous studies [48–51] also reported changes in purine metabolism, oxidative stress/redox homeostasis, energy metabolism, fatty acid metabolism, branched chain amino acids, phenylalanine and tyrosine metabolism, tryptophan metabolism, glycine derivation, and steroidogenesis in body fluids.

Growing evidence indicates that exosomes contribute to the progression of neurodegenerative disease [52]. Ghidoni et al. proposed the “Trojan horse” hypothesis of exosomes in neurodegeneration, a mechanism leading to the death of cells by shipping toxic agents in exosomes from cell to cell [53]. Indeed, many researchers believe that exosomes act as potential intercellular carriers of pathogenic proteins such as *α*-synuclein and cause impaired neuronal function [54]. In fact, a recent study by Han et al. showed that mice treated with serum exosomes from PD patients exhibited PD-relevant molecular, cellular, and behavioral phenotypes [55]. However, the opposing view is also considered feasible, that is, exosomes also offer neuroprotection, altering the abnormal cell's faulty programming by transmitting the “correct” information to the abnormal cell. There is no doubt that exosomes participate in the development process of PD, and the mechanism may derive from two aspects: first, exosomes are directly involved in information transmission and transport; second, exosomes indirectly affect biochemical reactions by altering the level of metabolites.

Based on the above mechanism, researchers have been studying and evaluating the feasibility of exosomes as therapeutic agents. Some studies have indicated that exosomes from different sources play a considerable role in protecting cells and alleviating the disease process. Research has revealed that exosomes from dental pulp stem cells inhibit 6-hydroxydopamine-induced apoptosis of dopaminergic neurons [56]. Similar findings indicated that exosomes from neurons, embryonic stem cells, neural progenitor cells, and astrocytes protect neurons [57]. Many studies have yielded similar results to the above, but they were based on exosomes of one cell type. It is undeniable that communication and transmission in the body generally involve the interaction of various types of cells. In this study, we focused on the blood-derived exosomes, which included exosomes derived from multiple cells rather than just one type of cell [58]. This setting allows our “therapeutics” to contain more comprehensive information. Exosomes released by different nerve cells carry different information and play different roles. Our study revealed that blood exosomes extracted from healthy volunteers have a neuroprotective effect on MPTP-treated mice. These protective effects were reflected by the restoration of the impaired motor coordination of mice, reduced loss of dopaminergic neurons, alleviated oxidative stress injury and neuroinflammation, and reduced cell apoptosis. We considered that the multiple neuroprotective effects in this study may be related to the source of exosomes, that is, blood exosomes are a complex composition comprising exosomes from a variety of nerve cells. There are two possible reasons for the therapeutic effects of blood exosomes. First, we can consider that some of the information carried by blood exosomes originates in the brain, for example, exosomes released by neurons cross the blood-brain barrier to the blood. Some of the information is consistent between the blood and the brain. Second, we speculate that intravenously injected exosomes entered the brain through the blood-brain barrier, altered the original proportion of exosomes in the brain, and successfully affected the physiological and pathological processes of the treated mice. The studies by Qu et al. [59] and Peter et al. [60] provide strong support for this inference. They found that intravenous or intranasal injection of exosomes was targeted in relevant brain regions. Our results have suggested that exosomes are a promising target for the treatment of PD.

At present, the diagnosis of PD mainly depends on the assessment of motor symptoms according to the UK Parkinson's Disease Society Brain Bank clinical diagnostic criteria [61] and the patient's response to dopaminergic drugs. The degeneration of dopaminergic neurons before the onset of PD symptoms lasts several decades [62]. Therefore, identifying biomarkers of preclinical PD is the key to treat and predict this disease and distinguish it from other diseases with the same manifestation. After years of failing to find available biomarkers in metabolites of body fluids such as blood or urine, the focus has turned to exosomes in the hope of gaining more useful information. Recent studies have suggested *α*-synuclein in plasma neuronal exosomes as a promising biomarker for the early diagnosis and prognosis of PD [63, 64]. In this study, widely-targeted metabolomics analysis was used to analyze the metabolite differences in exosomes in the serum and brain between healthy mice and MPTP-treated mice and explore the potential biomarkers in exosomes and the potential mechanism underlying exosome involvement in PD. Given the significant differences between MPTP mice and controls regarding the level of the 25 metabolites in the serum and brain, it was necessary to explore the potential of serum metabolites as biomarkers to differentiate between control and MPTP mice. We performed ROC curve analysis using the nine metabolites that contributed most to the differentiation of MPTP mice and controls. The data from our study suggested that nine exosomal metabolites, which significantly differed in both the brain and the serum between groups, exhibited excellent performance in differentiating PD and control mice. These metabolites included phenylacetate, (S)-(-)-2-hydroxyisocaproic acid, D-glucono-1,5-lactone, cholesterol, triethyl phosphate, 1,2-dichloroethane, 2,4,6-trimethylphenol, and terpinolene (AUC > 90%). Furthermore, 1,2-dichloroethane and (S)-(-)-2-hydroxyisocaproic acid have the ability to completely distinguish PD mice from control mice (AUC = 100%). Our study differs from previous reports in that we synchronously detected exosomal metabolites in the brain and serum. The advantage of this study was that the metabolites of serum exosomes could reflect the metabolites of brain exosomes, which can be difficult to acquire from clinical patients. Thus, these eight metabolites are potential clinical PD biomarkers.

However, the limitation of this study lies in the absence of a detailed mechanism for the treatment of PD by exosomes. Further research should focus on different metabolites or RNAs in exosomes, which can act as specific targets, to reveal the underlying mechanism of PD, provide feasible treatment options, and identify more accurate biomarkers.

## 5. Conclusion

In conclusion, through a series of experiments in behavior, pathology, and molecular biology, we confirmed that blood exosomes have protective effects on MPTP-treated PD model mice. This reflects the involvement of exosomes in the pathogenesis of neuron growth, oxidative stress, and neuroinflammation in PD. In addition, the widely-targeted metabolomics analysis revealed alteration of exosome metabolites and suggested the possibility of exosomes as potential biomarkers of PD. Our research provides novel suggestions for the treatment and prediction of PD.

## Figures and Tables

**Table 1:** 25 differentially expressed metabolites in serum and brain tissue.

Compounds	Class	Serum			Brain		
		VIP	*p* value	Trend	VIP	*p* value	Trend
(5-L-Glutamyl)-L-amino acid	Amino acid metabolomics	1.50329	0.0308	Up	1.016439	0.0183	Up
Dulcitol	Carbohydrate metabolomics	1.508731	0.0283	Down	1.931532	<0.0001	Down
Phenyllactate (Pla)	Organic acid and its derivatives	2.503092	<0.0001	Down	1.087977	0.0039	Down
9,10-DiHOME [(±)9,10-dihydroxy-12Z-octadecenoic acid]	Oxidized lipid	1.826174	0.0067	Up	1.367845	0.0027	Down
Cis-11,14,17-eicosatrienoic acid (C20:3)	Lipids fatty acids	1.56911	0.0203	Up	2.024657	<0.0001	Down
(S)-(-)-2-Hydroxyisocaproic acid	Organic acid and its derivatives	2.884665	<0.0001	Down	1.71456	0.0002	Down
Neopterin	Pteridines and derivatives	1.910464	0.005	Up	2.007469	<0.0001	Down
4-Ethylbenzoic acid	Benzene and substituted derivatives	1.945169	0.0041	Up	1.970055	<0.0001	Down
2-Picolinic acid	Pyridine and pyridine derivatives	1.332943	0.0481	Down	1.325756	0.0032	Down
Adenine	Nucleotide metabolomics	1.548782	0.0288	Down	1.323111	0.0064	Down
D-Glucono-1,5-lactone	Carbohydrate metabolomics	2.446655	<0.0001	Up	1.465692	0.0013	Up
Nicotinic acid	CoOthersEnzyme factor & vitamin	1.906224	0.0066	Down	1.084725	0.0124	Down
1-Naphthylacetic acid	Organic acid and its derivatives	1.490897	0.0181	Down	1.612702	0.0008	Up
Cholesterol	Lipids	2.80908	<0.0001	Up	2.226335	<0.0001	Up
Hexadecanamide	Lipids fatty acids	1.291087	0.0274	Up	1.304757	0.0301	Up
Triethyl phosphate	Organic acid and its derivatives	2.413266	0.0001	Down	2.140886	<0.0001	Down
1,2-Dichloroethane	Hydrocarbon derivative	2.825226	<0.0001	Up	1.319369	0.0062	Up
(E)-2-Octen-1-ol	Alcohol	1.769306	0.0077	Down	1.246376	0.0071	Down
Barbituric acid	Heterocyclic compound	1.554395	0.0277	Down	1.196766	0.0072	Down
Carene	Heterocyclic compound	2.232867	0.0004	Up	2.123199	<0.0001	Down
Hexyl acetate	Fatty acyls	1.79975	0.0087	Up	1.554381	0.0003	Down
2,4,6-Trimethylphenol	Phenols and its derivatives	2.660509	<0.0001	Down	1.641595	0.0002	Down
Terpinolene	Terpenoid	2.707682	<0.0001	Down	1.474065	0.0004	Down
Octanal	Aldehyde	1.481981	0.0278	Down	1.129016	0.0196	Down
Naphthalene	Benzene and substituted derivatives	1.663639	0.0121	Down	1.255286	0.0057	Down

The name of compounds, class, VIP, *p* value, and trend of metabolites were shown in the table.

## Data Availability

All data generated or analyzed during this study are included in the manuscript.

## Supplementary Materials

Supplementary materials Supplementary Figure 1: 2D and 3D PCA Plots indicated different metabolic characteristics in serum and brain tissue. Supplementary methods: the blood exosome isolation and validation methods. Supplementary Table 1: information of healthy volunteers. Supplementary Table 2: 69 differentially expressed metabolites in serum. Supplementary Table 3: 148 differentially expressed metabolites in the brain.
