# Supplementary material for: Blood Exosomes Have Neuroprotective Effects in a Mouse Model of Parkinson's Disease
Source: Oxid Med Cell Longev. 2020 Nov 26;2020:3807476. doi: 10.1155/2020/3807476 (PMC7714585; doi:10.1155/2020/3807476)
Supplement: Supplementary materials — Supplementary Figure 1: 2D and 3D PCA Plots indicated different metabolic characteristics in serum and brain tissue. Supplementary methods: the blood exosome isolation and validation methods. Supplementary Table 1: information of healthy volunteers. Supplementary Table 2: 69 differentially expressed metabolites in serum. Supplementary Table 3: 148 differentially expressed metabolites in the brain. [file 3807476.f1.pdf]

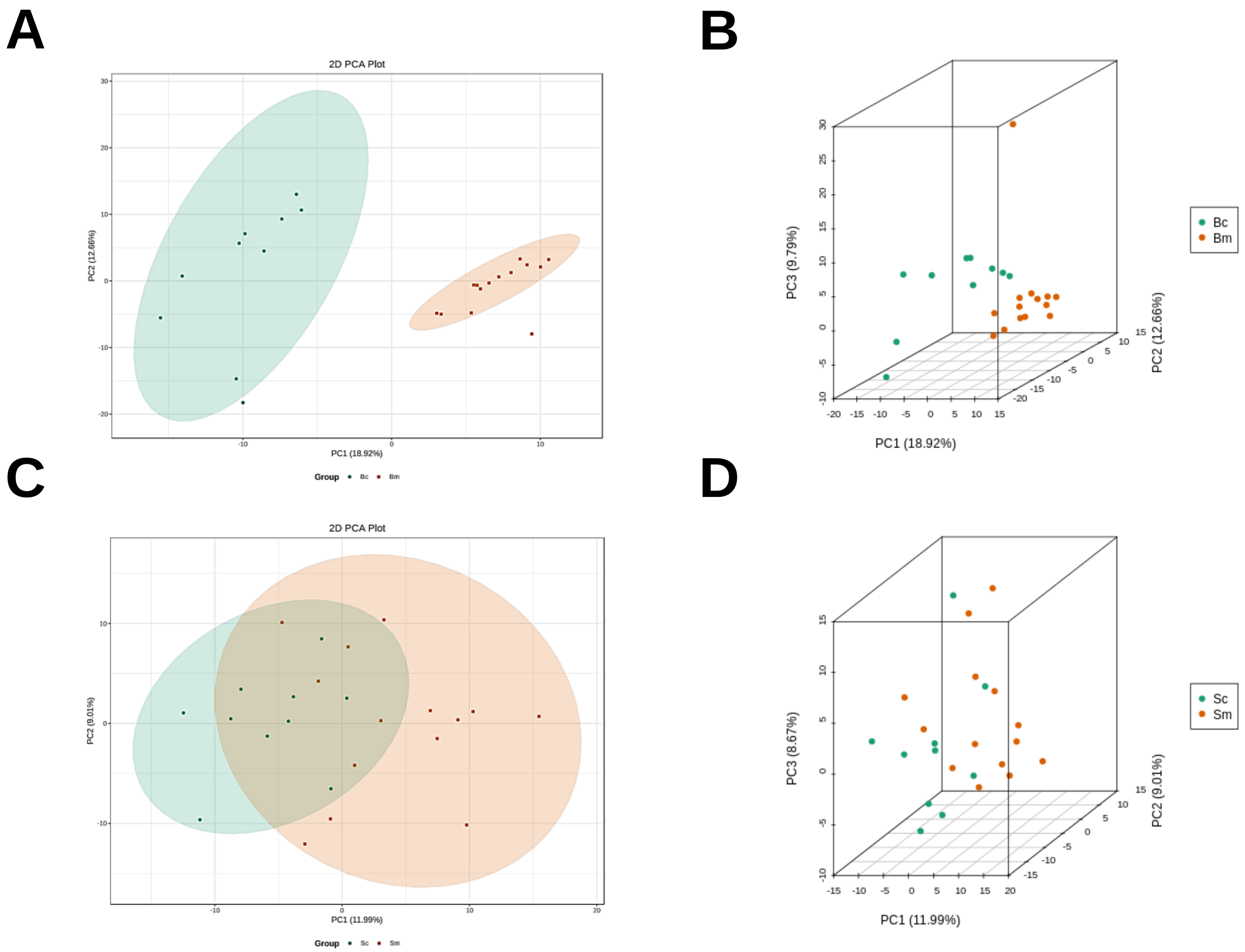

Supplementary Figure 1 2D and 3D PCA Plots indicated different metabolic characteristics in serum and brain tissue。 PCA results showed the trend of metabolome separation among the sample groups, and observed the variation between the difference groups and between the samples in the group, indicating that the 2 groups had different metabolic characteristics. Analysis of 14 PD mice and 10 control mice. 2D PCA Plot of metabolites in the brain (A) and serum (C), 3D PCA Plot of metabolites in the brain (B) and serum (D).

Supplementary methods

Sample preparation and extraction

Blood sample

Sample was thawed on ice, 3 volumes of ice-cold methanol was added to 1 volume of plasma/serum, whirl the mixture for 3 min and centrifuge it with 12,000 rpm at 4 ◦C for 10 min. Then collect the supernatant and centrifuge it at 12,000 rpm at 4 ◦C for 5 min. Finally collect the supernatant again and it will be used for LC-MS/MS analysis.

HPLC Conditions

The sample extracts were analyzed using an LC-ESI-MS/MS system (UPLC, Shim-pack UFLC SHIMADZUCBM A system, https://www.shimadzu.com/; MS, QTRAP® System, https://sciex.com/). The analytical conditions were as follows, UPLC: column, Waters ACQUITY UPLC HSS T3 C18 (1.8 µm, 2.1 mm*100 mm); column temperature, 40 ◦C; flow rate, 0.4 mL/min; injection volume, 2μL; solvent system, water (0.04% acetic acid): acetonitrile (0.04% acetic acid); gradient program, 95:5 V/V at 0 min, 5:95 V/V at 11.0 min, 5:95 V/V at 12.0 min, 95:5 V/V at 12.1 min, 95:5 V/V at 14.0 min.

ESI-QTRAP-MS/MS

LIT and triple quadrupole (QQQ) scans were acquired on a triple quadrupole-linear ion trap mass spectrometer (QTRAP), QTRAP® LC-MS/MS System, equipped with an ESI Turbo Ion-Spray interface, 30 operating in positive and negative ion mode and controlled by Analyst 1.6.3 software (Sciex). The ESI source operation parameters were as follows: source temperature 500 ◦C; ion spray voltage (IS) 5500 V (positive), -4500 V (negative); ion source gas I (GSI), gas II (GSII), curtain gas (CUR) were set at 55, 60, and 25.0 psi, respectively; the collision gas (CAD) was high. Instrument

tuning and mass calibration were performed with 10 and 100 μmol/L polypropylene glycol solutions in QQQ and LIT modes, respectively. A specific set of MRM transitions were monitored for each period according to the metabolites eluted within this period.

Supplementary Table 1 Information of Healthy volunteers

number	gender	age	Weight(kg)	Height(cm)
1	male	19	57	164
2	male	19	75	180
3	male	18	73	185
4	male	19	69	184
5	male	18	70	177
6	male	19	75	173
7	male	19	55	172
8	male	20	75	176
9	male	18	60	165
10	male	19	68	177

Supplementary Table 2 69 differentially expressed metabolites in serum

Compounds	Class	VIP	p-value (t-text)	trend
(5-L-Glutamyl)-L-Amino Acid	Amino Acid metabolomics	1.50329	0.0308	up
Allantoin	Organic Acid And Its Derivatives	1.645435	0.0206	up
N-Acetylneuraminic Acid	Amino Acid metabolomics	2.810086	<0.0001	down
Chenodeoxycholic Acid	Bile Acids	1.719309	0.0133	up
Dulcitol	Carbohydrate metabolomics	1.508731	0.0283	down
Phenyllactate (Pla)	Organic Acid And Its Derivatives	2.503092	<0.0001	down
Lysope 14:0	LipidsOthersPhospholipid	1.481949	0.0218	down
9,10-DiHOME [(±)9,10-dihydroxy-12Z-octadecenoic acid]	Oxidized lipid	1.826174	0.0067	up
Cis-11,14,17-Eicosatrienoic Acid (C20:3)	Lipids_Fatty Acids	1.56911	0.0203	up
(S)-(-)-2-Hydroxyisocaproic Acid	Organic Acid And Its Derivatives	2.884665	<0.0001	down
Neopterin	Pteridines and derivatives	1.910464	0.005	up
Thiamine Triphosphate	CoOthersEnzyme Factor &	1.879617	0.0046	down

	vitamin			
B-Hydroxypyruvic Acid	Organic Acid And Its Derivatives	1.445567	0.0405	up
Fumaric Acid	Amino Acid metabolomics	1.547576	0.0274	up
2-Deoxyribose 1-Phosphate	Carbohydrate metabolomics	1.709251	0.01	down
Formononetin	Carbohydrate metabolomics	1.414999	0.0402	up
Pyrophosphate	Organic Acid And Its Derivatives	2.516178	<0.0001	up
2,5-Furandicarboxylicacid	Organic Acid And Its Derivatives	1.617318	0.02	up
Methanesulfonic acid	Others	1.678012	0.0109	down
L-Asparagine Anhydrous	Amino Acid metabolomics	1.408834	0.0462	up
N-Acetyl-L-Glutamic Acid	Amino Acid metabolomics	1.713156	0.0124	down
P-Coumaraldehyde	Benzene and substituted derivatives	1.285275	0.0364	up
4-Ethylbenzoic Acid	Benzene and substituted derivatives	1.945169	0.0041	up
2-Picolinic Acid	Pyridine And Pyridine Derivatives	1.332943	0.0481	down
Acetylcholine Chloride	Cholines	2.392873	0.0001	down
1-Methylhistidine	Amino Acid metabolomics	1.675852	0.0148	down
3'-Aenylic Acid	Nucleotide metabolomics	1.853427	0.0036	up
Adenine	Nucleotide metabolomics	1.548782	0.0288	down
Cytidine	Nucleotide metabolomics	1.749622	0.0252	down
Epinephrine	Hormones	1.985023	0.0022	up
D-Galactose	Carbohydrate metabolomics	1.572895	0.0348	up
D-Glucono-1,5-Lactone	Carbohydrate metabolomics	2.446655	<0.0001	up
Nicotinic Acid	CoOthersEnzyme Factor & vitamin	1.906224	0.0066	down
1-Naphthylacetic Acid	Organic Acid And Its Derivatives	1.490897	0.0181	up
4-Guanidinobutyric Acid	Organic Acid And Its Derivatives	1.933636	0.0029	down
Azelaic Acid	Organic Acid And Its Derivatives	1.544053	0.0243	up
Creatinine	Organic Acid And Its Derivatives	1.71423	0.0096	down
Sebacate	Organic Acid And Its Derivatives	1.480941	0.0281	up
Acetaminophen Glucuronide	Carbohydrate metabolomics	1.224056	0.0384	up
H-Homoarg-Oh	Amino Acid metabolomics	1.439723	0.0425	down
Phosphoric Acid	Organic Acid And Its Derivatives	1.950611	0.0039	up
Cholesterol	Lipids	2.80908	<0.0001	up

2-Aminoadipic Acid	Organic Acid And Its Derivatives	1.347489	0.0482	down
Ergothioneine	Organic Acid And Its Derivatives	2.086389	0.0009	down
Hexadecanamide	Lipids_Fatty Acids	1.291087	0.0274	up
(-)-trans-Caryophyllene	Lipids_Fatty Acids	1.548686	0.0132	up
Triethyl phosphate	Organic Acid And Its Derivatives	2.413266	0.0001	down
2-Pyrrolidinone	Benzene and substituted derivatives	1.474133	0.0402	up
18-Hydroxycorticosterone	Lipids	2.63333	<0.0001	down
Dimethyl fumarate	Lipids_Fatty Acids	2.189748	0.0006	down
1,2-Dichloroethane	Hydrocarbon derivative	2.825226	<0.0001	up
Methyl tetradecanoate	Fatty acyls	1.463476	0.0436	down
Methyl?beta-D-galactopyranoside	Carbohydrate metabolomics	2.257974	0.0005	up
(E)-2-Octen-1-ol	Alcohol	1.769306	0.0077	down
3-Hydroxy-4,5-dimethyl-2(5H)-furanone	Heterocyclic compound	1.568378	0.0275	down
Methylisobutyrate	Organic Acid And Its Derivatives	1.436357	0.0286	down
N-Nitrosodiethylamine	Polyamine	1.428245	0.0218	down
Barbituric acid	Heterocyclic compound	1.554395	0.0277	down
carene	Heterocyclic compound	2.232867	0.0004	up
Hexyl?Acetate	Fatty acyls	1.79975	0.0087	up
2,4,6-Trimethylphenol	Phenols And Its Derivatives	2.660509	<0.0001	down
Terpinolene	Terpenoid	2.707682	<0.0001	down
alpha-Terpinene	Terpenoid	1.858838	0.0056	up
Octanal	Aldehyde	1.481981	0.0278	down
1-butanol	Alcohol	2.034277	0.0031	up
Naphthalene	Benzene and substituted derivatives	1.663639	0.0121	down
Nα-Acetyl-L-glutamine	Amino Acid metabolomics	1.469021	0.039	down
L-Glutamic acid	Amino Acid metabolomics	2.613966	<0.0001	down
Oleate	Lipids	1.217311	0.0405	up

Supplementary Table 3 148 differentially expressed metabolites in brain

Compounds	Class	VIP	p-value (t-text)	trend
(5-L-Glutamyl)-L-Amino Acid	Amino Acid metabolomics	1.0164394	0.0183	up
L-Cysteine	Amino Acid metabolomics	1.9618505	<0.0001	up
Benzoic Acid	Benzene and substituted	1.583751	0.0006	down

	derivatives			
Terephthalic Acid	Benzene and substituted derivatives	2.0292459	<0.0001	down
Tauroursodeoxycholic Acid	Bile Acids	1.7769221	<0.0001	down
Taurochenodesoxycholic Acid	Bile Acids	1.8423374	<0.0001	down
Phenethylamine	Polyamine	1.6678391	0.0002	down
Dulcitol	Carbohydrate metabolomics	1.9315324	<0.0001	down
Inosine	Nucleotide metabolomics	1.0822141	0.0204	down
3-Indolebutyric Acid	Indole And Its Derivatives	1.2947812	0.0063	down
Guanidinoethyl Sulfonate	Organic Acid And Its Derivatives	1.1777579	0.0179	down
Methylmalonic Acid	Organic Acid And Its Derivatives	1.0642954	0.0141	down
Phenyllactate (Pla)	Organic Acid And Its Derivatives	1.0879774	0.0039	down
Lysope 18:1	LipidsOthersPhospholipid	1.3555931	0.021	up
Lysope 18:0	LipidsOthersPhospholipid	1.5472096	0.002	up
Lysope 16:0	LipidsOthersPhospholipid	1.6538966	0.0002	up
9,10-DiHOME [(±)9,10-dihydroxy-12Z-octadecenoic acid]	Oxidized lipid	1.367845	0.0027	down
Palmitoleic Acid (C16:1)	Lipids_Fatty Acids	2.0376123	<0.0001	down
Hexadecanoic Acid (C16:0)	Lipids_Fatty Acids	1.7966922	<0.0001	down
Linoleic Acid (C18:2N6C)	Lipids_Fatty Acids	1.8432175	<0.0001	down
Dodecanoic Acid(C12:0)	Lipids_Fatty Acids	1.2862649	0.0102	down
Cis-11,14,17-Eicosatrienoic Acid (C20:3)	Lipids_Fatty Acids	2.0246571	<0.0001	down
(S)-(-)-2-Hydroxyisocaproic Acid	Organic Acid And Its Derivatives	1.7145603	0.0002	down
Neopterin	Pteridines and derivatives	2.0074686	<0.0001	down
Hypoxanthine-9-β-D-Arabinofuranoside	Nucleotide metabolomics	1.075642	0.0198	down
N-Acetylmethionine	Amino Acid metabolomics	1.0704085	0.0184	down
2-(4-Hydroxyphenyl)ethanol	Benzene and substituted derivatives	1.6027679	0.0006	down

cis-7-Hexadecenoic Acid	Lipids_Fatty Acids	1.672489	0.0004	down
cis-4-Hydroxy-D-proline	Organic Acid And Its Derivatives	1.1026618	0.0265	down
Glycerol 3-phosphate	Lipids_Fatty Acids	1.0627641	0.0458	down
Salicylic acid	Organic Acid And Its Derivatives	1.561779	0.0007	down
D-Arabinose 5-phosphate	Carbohydrate metabolomics	1.7115129	0.0003	down
Anthranilic acid	Benzene and substituted derivatives	1.9876917	<0.0001	down
Indoleacetaldehyde	Indole And Its Derivatives	1.3032201	0.002	down
D-Xylulose 5-phosphate	Carbohydrate metabolomics	1.4356481	0.0029	down
Vanillylmandelic acid	Benzene and substituted derivatives	1.2943917	0.005	down
PE(18:1(9Z)/0:0)	Lipids_Fatty Acids	1.3210476	0.0257	up
Sorbic acid	Organic Acid And Its Derivatives	1.1479028	0.0181	down
Decanal	Aldehyde	1.8000527	<0.0001	down
Methyl hexadecanoate	Fatty acyls	1.0311394	0.0458	down
4-tert-Octylphenol	Phenols And Its Derivatives	1.5341323	0.0003	up
PHENYL-BETA-D-GLUCOPYRANOSIDE	Carbohydrate metabolomics	1.5244368	0.001	down
2,4-Di-tert-butylphenol	Phenols And Its Derivatives	1.5008474	0.0007	up
Methylparaben	Benzoic Acid And Its Derivatives	1.889514	<0.0001	down
(±)4-HDHA [(±)4-hydroxy-5E,7Z,10Z,13Z,16Z,19Z-docosahexaenoic acid]	Oxidized lipid	1.0214453	0.0369	down
9-oxoODE [9-oxo-10E,12Z-octadecadienoic acid]	Oxidized lipid	1.6989341	<0.0001	down
Nonadecylic acid	Lipids_Fatty Acids	1.1886492	0.0216	up
N-Amidino-L-Aspartate	Amino Acid metabolomics	1.2933802	0.0029	down
Mono-Methyl Glutarate	Organic Acid And Its Derivatives	1.4123594	0.0027	down
L-Tyrosine	Amino Acid metabolomics	1.1898205	0.0044	down
L-Alanine	Amino Acid metabolomics	1.3159752	0.0021	down
L-Isoleucine	Amino Acid metabolomics	1.0373214	0.0138	down
L-Pyroglutamic Acid	Amino Acid metabolomics	1.2551514	0.0037	down
L-Valine	Amino Acid metabolomics	1.1797078	0.0097	down
Betaine	Alkaloid	1.0051541	0.0381	down

Phe-Phe	Amino Acid metabolomics	1.9414226	<0.0001	down
Methyleugenol	Benzene and substituted derivatives	1.1025906	0.0261	up
P-Coumaric Acid	Benzene and substituted derivatives	1.2419421	0.0035	down
2,5-Dihydroxy Benzoic Acid	Benzene and substituted derivatives	1.7688403	<0.0001	down
(3,4-Dimethoxyphenyl) Acetic Acid	Benzene and substituted derivatives	1.1182145	0.0119	down
4-Ethylbenzoic Acid	Benzene and substituted derivatives	1.9700551	<0.0001	down
Phthalic Acid	Benzene and substituted derivatives	1.0076797	0.0182	down
2-Picolinic Acid	Pyridine And Pyridine Derivatives	1.325756	0.0032	down
Choline	Cholines	1.1810585	0.0287	down
Adenine	Nucleotide metabolomics	1.3231114	0.0064	down
Adenosine	Nucleotide metabolomics	1.2286294	0.0098	down
Guanosine	Nucleotide metabolomics	1.2234202	0.0169	down
D-Sorbitol	Carbohydrate metabolomics	1.2278681	0.0078	down
D-Glucose	Carbohydrate metabolomics	1.5629465	0.0007	down
D-Glucono-1,5-Lactone	Carbohydrate metabolomics	1.4656916	0.0013	up
L-Ascorbate	CoOthersEnzyme Factor & vitamin	1.0642737	0.0466	down
Nicotinamide	CoOthersEnzyme Factor & vitamin	1.470342	0.0065	up
Nicotinic Acid	CoOthersEnzyme Factor & vitamin	1.0847247	0.0124	down
Indole-3-Acetic Acid	Indole And Its Derivatives	1.0119226	0.016	down
1-Naphthylacetic Acid	Organic Acid And Its Derivatives	1.612702	0.0008	up
6-Aminocaproic Acid	Organic Acid And Its Derivatives	1.1093408	0.0111	down
7-Methyluric Acid	Organic Acid And Its Derivatives	1.055454	0.0255	down
Homovanillic Acid	Organic Acid And Its Derivatives	1.1525094	0.0057	down
Hydrocinnamic Acid	Organic Acid And Its Derivatives	1.7117998	<0.0001	up
Lysopc 16:0	LipidsOthersPhospholipid	1.1967527	0.0378	up
Lysopc 18:0	LipidsOthersPhospholipid	1.3642159	0.0163	up
4-Hydroxybenzaldehyde	Benzene and substituted derivatives	1.1608982	0.0049	down

Taurocholic Acid Sodium Salt Hydrate	Organic Acid And Its Derivatives	1.4287535	0.0015	up
2-(Formylamino)Benzoic Acid	Benzene and substituted derivatives	1.001325	0.0189	down
N-Acetylglycine	Amino Acid metabolomics	1.1129735	0.0158	down
7-Methylguanine	Nucleotide metabolomics	1.012108	0.0174	down
Pantetheine	CoOthersEnzyme Factor & vitamin	1.5871872	<0.0001	down
5'-Deoxy-5'-(Methylthio) Adenosine	Nucleotide metabolomics	1.3988769	0.0023	down
Trans-Citridic Acid	Organic Acid And Its Derivatives	1.6743321	0.0001	down
6-Phosphogluconic Acid Trisodium Salt	Carbohydrate metabolomics	1.2538245	0.0158	up
Cholesterol	Lipids	2.2263354	<0.0001	up
Indole	Indole And Its Derivatives	1.4597394	0.0027	up
Piperidine	Benzene and substituted derivatives	1.0601314	0.0089	down
2-Methylguanosine	Nucleotide metabolomics	1.0686127	0.0102	down
2-Hydroxycinnamic acid	Benzene and substituted derivatives	1.1314423	0.0064	down
DL-o-Tyrosine	Organic Acid And Its Derivatives	1.1758803	0.0051	down
Hexadecanamide	Lipids_Fatty Acids	1.3047565	0.0301	up
N-Acetyl-L-alanine	Organic Acid And Its Derivatives	1.7793308	<0.0001	down
Triethyl phosphate	Organic Acid And Its Derivatives	2.1408865	<0.0001	down
PAF C-16	Lipids	1.4522904	0.0074	up
alpha-Cadinene	Lipids_Fatty Acids	1.4098875	0.0014	down
2'-Hydroxy-5'-methylacetophenone	Benzene and substituted derivatives	1.3144818	0.005	up
2-(3,4-dimethoxyphenyl)ethanamine	Benzene and substituted derivatives	1.1648478	0.005	down
beta-Cubebene	Lipids_Fatty Acids	1.3413787	0.0014	down
Benzylcinnamate	Benzene and substituted derivatives	1.0589763	0.0169	up
Isoamyl butyrate	Fatty acyls	1.1725654	0.0177	down
Ethyl dodecanoate	Fatty acyls	2.0857838	<0.0001	up
Propylpropionate	Fatty acyls	1.0867356	0.0269	down
1,2-Dichloroethane	Hydrocarbon derivative	1.3193694	0.0062	up

Cyclohexanone	Ketones	1.8072582	<0.0001	down
Butyl 3-methylbutanoate	Fatty acyls	1.049239	0.0452	down
Ethyl decanoate	Fatty acyls	1.8440143	<0.0001	down
2,2,2-Trichloroethanol	Alcohol	1.3925061	0.0032	down
Methylcysteine	Amino Acid metabolomics	1.2062025	0.0044	down
Benzophenone	Benzene and substituted derivatives	1.040007	0.037	down
TranexamicAcid	Organic Acid And Its Derivatives	1.26248	0.0198	up
(E)-2-Octen-1-ol	Alcohol	1.2463765	0.0071	down
2-Pentadecanone	Ketones	1.899365	<0.0001	up
Methyl dihydrojasmonate	Organic Acid And Its Derivatives	1.8285179	<0.0001	up
3,5-Dinitrosalicylic acid	Organic Acid And Its Derivatives	1.5016205	0.001	up
3-Methylthiophene	Heterocyclic compound	1.7098025	0.0001	down
Barbituric acid	Heterocyclic compound	1.1967662	0.0072	down
2,4-Dichlorophenoxyacetic acid	Benzene and substituted derivatives	2.1033977	<0.0001	up
carene	Heterocyclic compound	2.123199	<0.0001	down
Hexyl?Acetate	Fatty acyls	1.554381	0.0003	down
Farnesol	Alcohol	1.0594597	0.0177	down
2,4,6-Trimethylphenol	Phenols And Its Derivatives	1.6415951	0.0002	down
Terpinolene	Terpenoid	1.4740649	0.0004	down
(+)-borneol	Alcohol	2.0544875	<0.0001	down
p-Menth-1-en-4-ol	Terpenoid	1.8839988	<0.0001	down
2-Pentyl-3-phenyl-2-propenal	Aldehyde	2.122818	<0.0001	up
Octanal	Aldehyde	1.1290156	0.0196	down
Pyrene	Benzene and substituted derivatives	2.1075731	<0.0001	up
MESITYL OXIDE	Ketones	1.2746507	0.008	down
METHYL VALERATE	Fatty acyls	1.8928732	<0.0001	down
1-PENTADECANOL	Alcohol	1.7215666	<0.0001	up
DIISOBUTYL PHTHALATE	Benzene and substituted derivatives	1.3033899	0.0032	down
Dibutyl phthalate	Benzene and substituted derivatives	1.0487835	0.0207	down
Naphthalene	Benzene and substituted derivatives	1.2552856	0.0057	down
ETHYL ISOBUTYRATE	Fatty acyls	1.2435101	0.0083	down
Choline chloride	Others	1.1436045	0.0056	down

caprate (10:0)	Lipids	1.4901922	0.0018	down
Isobutyl decanoate	Fatty acyls	1.838032	<0.0001	up
proline betaine	Organic Acid And Its Derivatives	1.1284421	0.0237	down
Bisphenol propane	Benzene and substituted derivatives	2.1486378	<0.0001	up
N-methylalanine	Amino Acid metabolomics	1.052861	0.022	down
2'-Deoxycytidine 5'-Diphosphate	Nucleotide metabolomics	1.286858	0.0183	up
Dethiobiotin	Others	1.81189	<0.0001	down
